# Yang-xin-xue keli exerts therapeutic effects *via* regulating mitochondrial homeostasis and function in doxorubicin-induced rat heart failure

**DOI:** 10.3389/fphar.2022.931453

**Published:** 2022-08-30

**Authors:** Kunlan Long, Ziyi Zhao, Jun Chen, Lijia Zhi, Chunxia Wang, Dan Liao, Meng Wang, Peiyang Gao

**Affiliations:** ^1^ Intensive Care Unit, Hospital of Chengdu University of Traditional Chinese Medicine, Chengdu, China; ^2^ TCM Regulating Metabolic Diseases Key Laboratory of Sichuan Province, Hospital of Chengdu University of Traditional Chinese Medicine, Chengdu, China

**Keywords:** yangxinxue keli (YXXKL), heart failure, mitochondrial homeostasis, reactive oxygen species (ROS), oxidative stress

## Abstract

**Background:** Heart failure, especially chronic heart failure, is generally induced by the accumulation of reactive oxygen species (ROS), as well as the subsequent loss of mitochondrial permeability transition pore (mPTP) openings and pathological mitochondrial dysfunction. Herein, we explored the therapeutic effects of the Chinese medicine Yangxin Keli (YXXKL) on chronic heart failure and its underlying working mechanism.

**Methods:** To mimic oxidative stress-induced chronic heart failure, a rat heart failure model was induced by the administration of DOX. Transthoracic echocardiography was performed to confirm the successful establishment of the heart failure model by observing significantly decreased cardiac function in the rats. Mitochondrial membrane potential, function, and ATP synthesis activity were measured after YXXKL was employed.

**Results** The administration of YXXKL not only significantly improved cardiac function but also reversed the myocardium loss and fibrosis induced via DOX. Moreover, the administration of YXXKL also increased ATP synthesis and mitochondrial DNA mass in left ventricular tissues, which indicated that mitochondria may be a key target of YXXKL. Thus, we employed rat cardiomyocyte H9c2 and primary rat cardiac myocytes (RCMs) to induce oxidative stress-induced myocardial injury via DOX treatment. YXXKL-medicated serum promoted cell proliferation, which was inhibited by the addition of IC30 DOX, and the serum also inhibited cell apoptosis, which was promoted by the addition of IC50 DOX. YXKL-medicated serum was able to scavenge ROS and maintain the mitochondrial membrane potential as well as promote mitochondrial function, including the promotion of ATP synthesis, mitochondrial DNA mass, and transcriptional activity. Furthermore, we also observed that YXXKL-medicated serum inhibited DOX-induced autophagy/mitophagy by scavenging ROS.

**Conclusion:** Taken together, we conclude that YXXKLI may exert therapeutic effects on oxidative stress-related heart failure via the regulation of mitochondria.

## Introduction

Chronic heart failure (CHF) is a common clinical syndrome and is pathologically indicated by significant morbidity and mortality (McMurray and Stewart, 2000). Worldwide, over 26 million patients suffer from CHF, which has resulted in a low quality of life for these patients (Bui et al., 2011). These patients are recommended to take their medications, monitor their symptoms, seek help when needed, eat and drink in a healthy manner, and manage depression (National Clinical Guideline Centre (UK), 2010; McMurray et al., 2012; Tu et al., 2014). Additionally, the maintenance and improvement of physical fitness can critically promote the prognosis of heart failure. CHF is characterized by interstitial fibrosis, chamber remodeling, and reduced ventricular compliance (Moore-Morris et al., 2014). During the long-term processes of CHF, extracellular matrix (ECM) proteins, including collagen type I and fibronectin, have been observed to be extensively produced by activated CFs (Moore-Morris et al., 2014).

Doxorubicin (DOX) is an anthracycline that was initially extracted and identified from *Streptomyces pneumoniae*. Due to its cytotoxicity, DOX has been used for the treatment of several cancers, including breast cancer, lung cancer, gastric cancer, ovarian cancer, and Hodgkin’s lymphoma (Arcamone et al., 1969; Cortes-Funesand and Coronado, 2007). Unfortunately, DOX is also considered to be one of the most cardiotoxic medications in clinical use (Singal et al., 1997). Thus, DOX-induced cardiotoxicity is one of the side effects of the medications used to treat CHF. The most likely mechanism of its cardiotoxicity is that DOX causes increased production of reactive oxygen species (ROS), thus leading to damage to DNA, proteins, and lipids, as well as the loss of mitochondrial homeostasis and function, which ultimately causes the death and dysfunction of cardiomyocytes (Kim et al., 2006; Zhang et al., 2012).

Chinese herbal medicine (TCM) has been developed and clinically used for more than 2,000 years in China, with over 70% of patients choosing TCM as a treatment (Chen and Lu, 2006). TCM has been reported to be an efficient strategy to manage CHF because it represents a simple, inexpensive, and noninvasive approach for the evaluation of the severity of HF (Li et al., 2013; Fu et al., 2016). It has been reported that Shensong Yangxin capsules, a combined herbal formulation widely used in China for the treatment of arrhythmias, have the benefits of suppressing ventricular premature complexes (VPCs) and improving cardiac function (Wang X. et al., 2017). Yang et al. (2019) assessed the efficacy and safety of Fuzi formulae (FZF) in treating CHF, and they demonstrated that FZF exerts efficacy and additional benefits on CHF. It has also been reported that gallic acid improves cardiac dysfunction and fibrosis in pressure overload-induced heart failure (Jin et al., 2018), and it can produce benefits for vascular calcification (Kee et al., 2014), cardiac hypertrophy, and fibrosis (Ryu et al., 2016), hypertension (Jin et al., 2017a), and oxidative stress (Jin et al., 2017b).

“Yangxinxue keli” (YXX), which is a traditional Chinese medicine made from Bupleurum Chinense, Concha Ostreae, Angelica Sinensis, Ligusticum chuanxiong Hort, Radix Rehmanniae, Paeonia lactiflora, Acanthopanax giraldii Hams, Semen zizyphi spinosae, sun-dried ginseng, Polygonatum odoratum, and Cinnamomum cassia Presl, has been widely used to clinically treat CHF. YXX is one of the main treatments for cardiovascular disease in the field of Chinese medicine. In this study, we focused on the evaluation of the efficacy and effects of YXX in the treatment of CHF. Furthermore, we also investigated the effects of YXX on the oxidative stress-induced loss of mitochondrial homeostasis and function and provided the molecular basis for its use as a medication for the treatment of CHF.

## Materials and methods

Preparation of Yang-Xin-Xue-Ke-Li agent (YXX keli), an extract from 11 species of medical herbs: 7.84 g of Bupleurum Chinense, 7.84 g of Concha Ostreae, 7.84 g of Angelica Sinensis, 4.71 g of Ligusticum chuanxiong Hort, 6.27 g of Radix Rehmanniae, 4.71 g of Paeonia lactiflora, 15.68 g of Acanthopanax giraldii Hams, 9.42 of Semen zizyphi spinosae, 6.27 g of sun-dried ginseng, 6.27 g of Polygonatum odoratum, and 3.13 g of Cinnamomum cassia Presl, was prepared by the Pharmacy Department of the Hospital of Chengdu University of Traditional Chinese Medicine. In brief, the abovementioned materials were concerted in a ratio of 5:5:5:3:4:3:10:6:4:4:2. All the herbs were placed in a container and soaked in 1,000 ml of water for 1 h. First, we used intense fire to heat the herbs and water until it was boiled and then turned the fire to slow fire for 60 min; the liquid was then poured out to another container. Second, we added 750 ml water to the first container and then decocted the herbs using intense fire for 30 min; the liquid was also poured out. Third, we mixed the liquid obtained from the first and second steps, which was concentrated on a drug solution containing 0.4 g raw herb per mL, and stored at 4°C.

**FIGURE 1:**
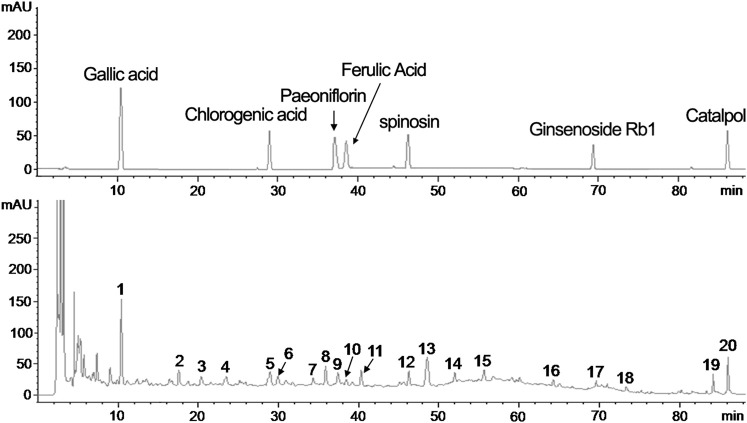
The fingerprint of the YXX keli by HPLC. **(A)**. The reference standard. **(B)**. The 20 characteristic peaks of the YXX keli fingerprint. The No. 1 peak (Gallic acid), No. 5 peak (Chlorogenic acid), No. 8 peak (Paeoniflorin), No. 9 peak (Ferulic Acid), No. 12 peak (Spinosin), No. 17 peak (Ginsenoside Rb1), and No. 20 peak (Catalpol) are determined compared with the reference standard.

The fingerprint of the YXX keli by HPLC (Agilent Technologies 1,200 Series) chromatography was carried out for quality control. As shown in Figure 1, the fingerprint of YXX keli is composed of 20 characteristic peaks. Peaks 1, 6, 8, 10, 11, 13, 17, and 20 are Gallic acid, Chlorogenic acid, Paeoniflorin, Ferulic Acid, Spinosin, Ginsenoside Rb1, and Catalpol compared with the reference standard, respectively. Especially, the No. 1 peak (Gallic acid) is used as the quality control and reference peak by its properties of good separation, no impurity interference, and relative stability.

Experimental protocol: all the animal experiments were conducted according to the Institutional Animal Care and Use Committee of Institute of Chengdu University of Traditional Chinese Medicine. Six-week-old female SD rats were purchased from Dashuo Experimental Animal Company (Chengdu, China) and raised in the SPF animal facilities. Rats were randomly divided into six groups: 1) the control group (Untreated, *n* = 12, intragastrically administrated 0.5 ml of PBS per day); 2) the DOX-treated heart failure group (Model, *n* = 13, intragastrically administrated 0.5 ml of PBS per day); 3) the low-dose group (LD, *n* = 12, intragastrically administrated 0.25 ml of YXX keli per day); 4) the middle-dose group (MD, *n* = 17, intragastrically administrated 0.5 ml of YXX keli per day); 5) the high-dose group (HD, *n* = 14, intragastrically administrated 1 ml of YXX keli per day); and 6) Benazepril group (Benazepril, *n* = 14, intragastrically administrated 0.66 mg per day) for 4 weeks. Briefly, for the low-dose group, 1.25 g/kg/day of the drug was administrated; for the middle-dose group, 2.5 g/kg/day of the drug was administrated; for the high-dose group, 5 g/kg/day of the drug was administrated.

After all administration, the cardiac function of the rats was evaluated by transthoracic echocardiography. All rats were weighed and anesthetized with 3% isoflurane. Then, left ventricular ejection fraction (EF), stroke volumes (SV), cardiac output (CO), and fractional shortening were measured and qualified using a Vevo 2,100 high-resolution imaging system equipped with a transducer with center frequencies ranging from 13 to 24 MHz (MS250; Visual Sonics, Toronto, Canada).

HE staining: the collected tissues were fixed with 4% paraformaldehyde solution, dehydrated with alcohol, and cleared by xylene, followed by paraffin-embedding. Next, the paraffin-embedded sections were dewaxed with xylene twice for 10 min each time, dehydrated with gradient alcohol, and washed under running water. The sections were stained with hematoxylin for 5 min, added with 0.5% alcohol hydrochloric acid, and returned to blue for 30 s with the addition of 0.5% ammonia. Finally, the sections were stained with 0.5% eosin solution for 2 min, dehydrated with ethanol, cleared with xylene, and sealed using neutral gum. The pathological changes were observed under a microscope.

Masson staining: the paraffin-embedded sections (3–4 μm thick) were dewaxed, stained with Harris hematoxylin for 3 min, differentiated by 1% hydrochloric ethanol for 3–5 s, and returned to blue for 1 min in warm water. Then, the sections were stained with ponceau-acid fuchsin for 3 min, immersed in 2% glacial acetic acid solution for 1 min, and differentiated by 1% molybdophosphoric acid for 1 min, followed by the removal of excessive molybdophosphoric acid. Subsequently, the sections were counterstained with 2% aniline blue for 1 min, immersed in 0.2% glacial acetic acid solution for 1 min, and washed with 95% alcohol. Finally, the sections were dehydrated with gradient alcohol, cleared with xylene, sealed with neutral gum, and observed under a microscope.

ANP and BNP detection: after animal experiments, a 2-ml blood sample for each rat was harvested into a chilled vacutainer tube (Beckton-Dickinson Co., Rutherford, NJ) supplemented with EDTA and aprotinin. The plasma was then obtained by centrifuging at 5,000 g for 10 min at 4°C and stored at −80°C. To detect atrial natriuretic peptide (ANP) in plasma, the stored sample was detected by using a commercial kit (Peninsula Laboratories Inc., Belmont, CA). Enzyme-linked immunosorbent assay (ELISA) was employed to detect B-type brain natriuretic peptide (BNP) in plasma. Levels of BNP were determined and calculated according to the kit instructions.

Cell culture: the rat heart-derived myoblast cell line H9c2 was bought from the American Type Culture Collection (ATCC; cat. No.: CRL-1446). Cells were maintained in the Dulbecco’s modified Eagle’s medium (DMEM) supplement with 10% fetal bovine serum (FBS) and 100 U/ml of penicillin and 100 μg/ml of streptomycin (Gibco) at 37°C in a humidified atmosphere with 5% CO2.

Neonatal rat cardiac myocytes (RCM) were isolated from the Sprague–Dawley rats on postnatal days 1–2, using SoniConvert^®^ Single Cell Suspension Preparation System (DocSense, Chengdu, China) by following the manufacturer’s instructions. Cells were cultured in Minimum Essential Medium (MEM)-α containing 10% FBS, 100 U/ml of penicillin, 100 μg/ml of streptomycin (Gibco), and 100 μmol/L boromodeoxyuridine (Sigma). At 48 h after plating, the culture media was replaced with 0.5% FBS MEM-α.

To evaluate the effects of DOX on proliferation in H9c2, 1 μmol/L DOX was supplemented into the medium for 24-h incubation followed by analysis. To evaluate the effects of DOX on promoting apoptosis or inhibiting mitochondrial function in RCM, 1.54 μmol/L DOX was supplemented into a medium for 24-h incubation followed by analysis.

ATP measurement: the cells were plated in 96-well plates at a density of 5×10^4^ cells per well. ATP-dependent luciferase luminescence was measured in cells incubated using the CellTiterGlo kit according to the manufacturer’s instructions.

Quantitative PCR to detect mtATP6 and r18S: QuantiFast SYBR Green PCR kit was employed to detect the mtATP6 and r18S according to the manufacturer’s instructions. The PCR amplification was carried out in the conditions as followed: one cycle of 94°C for 5 min, 30 cycles of 94°C for 30 s, 60°C for 30 s, and then one cycle at 60°C for 2 min, and hold at 4°C. Primer sequences and their corresponding PCR product for mtATP6 and r18S were as follows: mtATP6 forward, 5′-ATG​AAC​GAA​AAT​CTA​TTT​GCC​TC-3′ and reverse 5′-TTA​TGT​ATT​ATC​ATG​TAG​ATA​TAG​GCT​TAC​TAG​GA-3’; r18S forward 5′-AGG​ACT​CTT​TCG​AGG​CCC​TGT​AAT​TGG-3′ and reverse 5′-TTC​ACC​TCT​AGC​GGC​GCA​ATA​CGA​ATG-3’.

CCK-8 assay: the Cell Counting Kit-8 (CCK-8) was used to determine the viability of the cells according to the instructions of the manufacturer. Firstly, the cells were plated in 96-well plates at a density of 5 × 103 cells per well. Then, the cells treated with 10ul of CCK-8 solution at the indicated time periods. After incubated in the dark for 2 h, the absorbance of the cells was measured at a wavelength of 450 nm.

PI staining followed by flow cytometry: cells were suspended using 0.25% trypsin (Thermo Scientific, Waltham, MA, United States) and washed with PBS three times. At the last wash, the cell pellet was suspended and fixed in 70% ice-cold alcohol overnight at 4°C. Then cells were washed with ice-cold PBS three times and suspended with 400 μl PI solution (5 μg/ml) for 30 min in the dark. Then, cells were analyzed by flow cytometry using three laser Navios flow cytometers (Beckman Coulter, Brea, CA, United States).

EdU staining: EdU assay was used to determine the cell proliferative capacity. The cells in a logarithmic state were counted and adjusted to 1×10^6^ cells/mL, Then, cells were seeded at a density of 5×10^3^ cells per well in 96-well plates. The newly synthesized DNA of the cells was assessed by the EdU incorporation assays according to the instructions. Fluorescence microscopy was used to acquire and analyze the EdU incorporation rate.

Apoptosis analysis: cells were treated with hyperthermia as described previously for 2 and 4 h at 42°C. The Annexin V-FITC/PI double staining was performed according to the manual of the Annexin V-FITC/PI apoptosis detection kit (Life Technologies, Grand Island, NY, US). Approximately 5×10^5^ cells were collected, washed with chilled PBS, and resuspended in the binding buffer containing 5 μl Annexin V-FITC for 10 min’s incubation in the dark at room temperature, and then the binding buffer was removed by centrifugation at 1000g/4°C for 10 min. The cells were resuspended in a reaction buffer containing 5 μl PI. Then, flow cytometric analysis was immediately performed to detect apoptosis.

Quantitative ROS measurement: the cells were labeled with the fluorescent DCFH-DA probe to determine the intracellular ROS generation and measured with a flow cytometer. The cells were incubated with DCFH-DA at 37°C for 30 min and harvested. After that, the cells estimated the intracellular ROS by flow cytometry. The cells exhibiting positive FITC fluorescence were indicative of intracellular ROS generation.

JC-1 staining (determination of mitochondrial membrane potential): A JC-1 staining assay kit was used to determine the alteration of mitochondrial membrane potential in the cells according to the instructions. Briefly, 1×10^6^ cells were washed with PBS and stained with 20 ug/ml JC-1 at 37°C for 30 min in the dark. After washing twice with staining buffer, the cells were detected by flow cytometry.

RT-qPCR analysis: TRIZol reagent was used to extract total RNA from the cells according to the manufacturer’s instructions. 2ug RNA was conducted to reverse transcription using a first strand cDNA synthesis kit. Real-time PCR was performed using the Applied Biosystems 7,500 Fast Real-time PCR System instrument and software. The expression of target genes was calculated based on formula 2^-△△Ct^.

Transmission electron microscopy (TEM) to detect autophagosomes: the cells were washed with 0.1 M cacodylate buffer (pH 7.3) and fixed with PBS which contains 3% glutaraldehyde and 2% paraformaldehyde. The rest of the procedure was conducted according to the standard protocol. After that, the cells were observed under Transmission Electron Microscope (JEOL JEM1200^®^ EX-II).

Western blot to detect PINK1, Parkin, and Lc3I/II: the cells were washed twice with cold PBS and lysed in RIPA buffer. The protein concentrations were measured by BCA protein assays. For each sample, the same amount of protein was loaded onto 10% and 12.5% SDS-PAGE, which was then electro-transferred to the polyvinylidene difluoride (PVDF) membrane. Blots were probed with antibodies against PINK1, Parkin, Lc3I, and Lc3IIfor overnight at 4°C, and then incubated with the relevant secondary antibodies for 1 h at 37°C. Western blot images were identified using a FluorChem E System.

Statistical analysis: all of the data were calculated and expressed as the mean ± SD. The differences between the two groups were tested by a *t*-test. The differences among the groups were performed using a one-way analysis of variance (ANOVA). Only when a significant difference was determined by the ANOVA, multiple-comparison tests were applied. Values of *p* < 0.05 were considered to be statistically significant.

## Results

**FIGURE 2:**
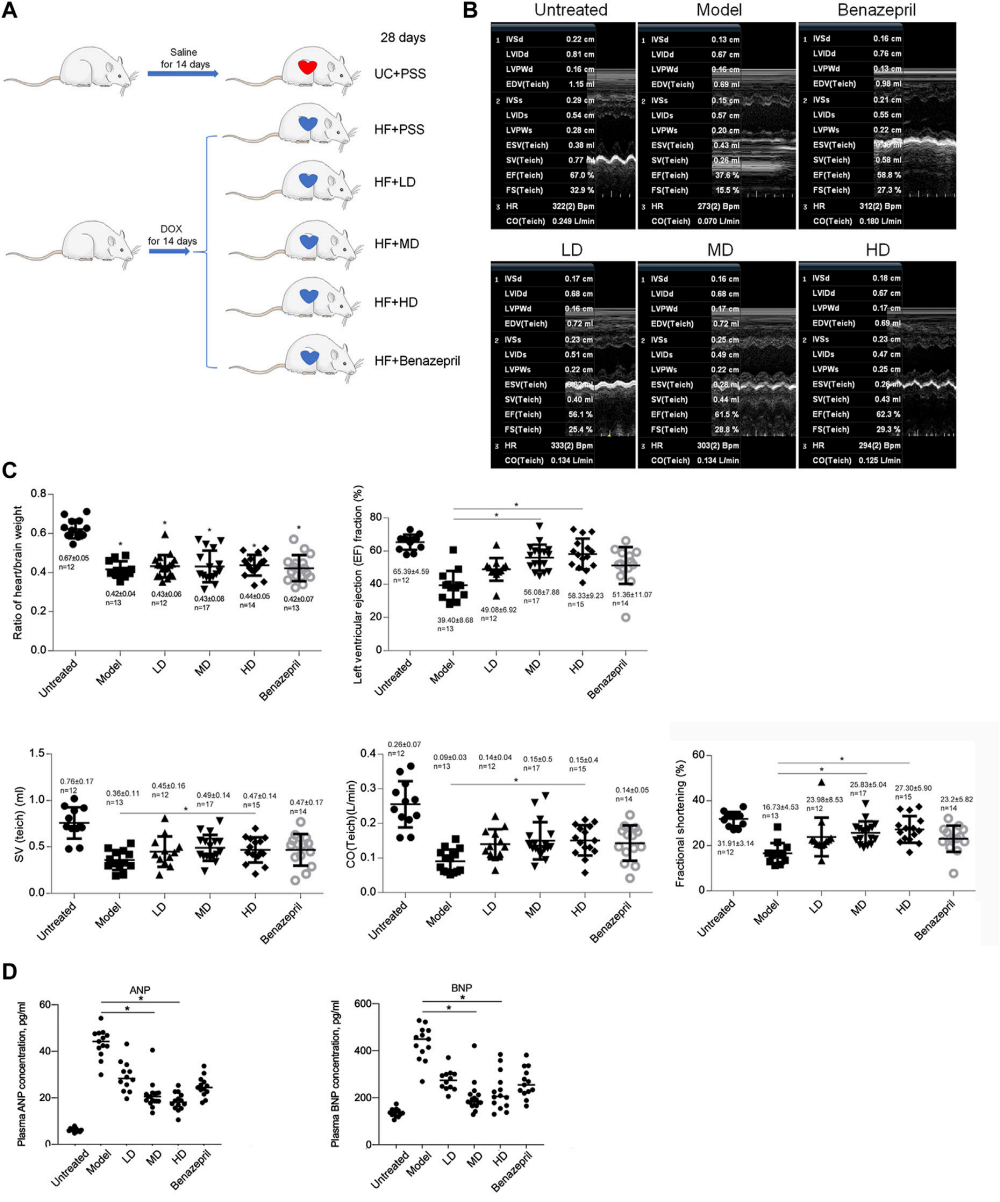
Model building and transthoracic echocardiography to evaluate the cardiac function of rats. **(A)** building of heart failure of a rat model. UC + PBS: untreated group fed with the same volume of PBS; HF + PBS: heart failure group fed with the same volume of PBS; HF + LD: heart failure group fed with a low dose of YXX keli; HF + MD: heart failure group fed with a middle dose of YXX keli; HF + HD: heart failure group fed with a high dose of YXX keli; HF + Benazepril: heart failure group fed with Benazepril. **(B)**. Transthoracic echocardiography was performed after all experiments. **(C)**. In different groups, cardiac function was performed. *p < 0.05, vs. Model group. **(D)**. ANP and BNP in different groups were measured. *p < 0.05, vs. Model group.

Establishment of a heart failure rat model: after DOX injection by following a detailed procedure (Figure 2A), the Mock group (Mock) and model group (Model) were analyzed for confirming the successful model establishment. After a 28-day animal experiment, all rats survived. The ratio of heart/brain weights was comparable among Mock and Model. After modeling, the ratio of heart/brain decreased significantly in the Model group than in the Mock group (0.67 ± 0.05 g vs. 0.42 ± 0.04g, *p* < 0.05, Figure 2C), indicating the decreasing weight of the heart. The cardiac function of the rats was tested at the end of 28 days after DOX injection (Figure 2B). Compared with the Mock group, Left ventricular ejection fraction (LVEF), SV (Teich), CO (Teich) and fractional shortening (FS) decreased significantly (LVEF: 39.40 ± 8.68% vs. 65.39 ± 4.59%, *p* < 0.05; SV: 0.36 ± 0.11 ml vs. 0.76 ± 0.17 ml, *p* < 0.05, CO: 0.09 ± 0.03 L/min vs. 0.26 ± 0.07 L/min, *p* < 0.05; FS: 16.73 ± 4.53% vs. 31.91 ± 3.14%, *p* < 0.05). Taken together, 14 days of administration of DOX significantly decreased heart weight, LVEF, SV, CO, and FS, demonstrating a significant decrease in heart function and successful establishment of heart failure.

Dose-dependent effects of YXX keli on heart failure: after 28 days of administration of YXX keli with different doses (low-dose group, LD; middle-dose group, MD; and high-dose group, HD), we measured heart/brain weight ratio and cardiac function by employing transthoracic echocardiography (Figures 2B,C). Administration of LD, MD, HD, and Benazepril, which was used as a positive control for intervention presented no significant differences in the heart/brain weight ratio, LVEF, SV, CO, and FS. Compared with the model group, MD, HD, and Benazepril groups, but not the LD group, significantly increased LVEF, SV, CO, and FS. Furthermore, we also detected the effects of YXX keli on ANP and BNP levels in plasma. As is presented in Figure 2D, compared to the model group, both ANP and BNP levels decreased significantly in MD and HD groups. These data indicated the promoting effect of MD and HD of YXX keli on cardiac function.

**FIGURE 3:**
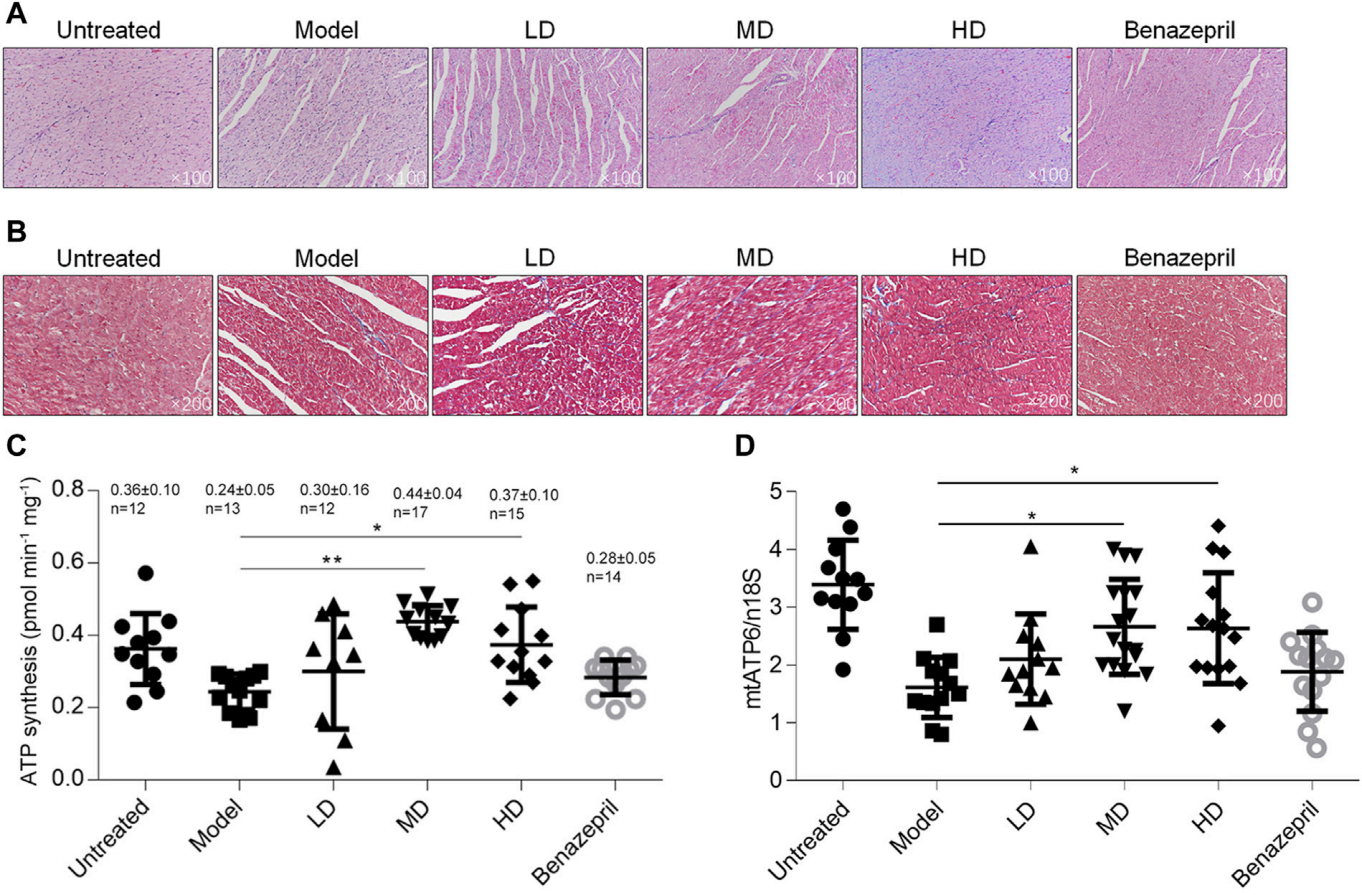
Detection of heart injury and metabolic function after the YXX keli treatment. To evaluate the myocardial injury, H&E **(A)** and Masson’s Trichrome **(B)** stained sections was performed. After being sacrificed, the left ventricle was freshly prepared and detected for ATP synthesis **(C)** and mitochondrial DNA mass **(D)**. *p < 0.05, vs. model group; **p < 0.01, vs. model group.

To access whether YXX keli improved myocardial injury after heart failure, histological analysis of H&E and Masson’s Trichrome stained sections was performed. As shown in Figures 3A,B, heart failure caused loss of myocardium and local necrosis in the model group compared with a mock group (Figure 3A), and extensive areas of fibrosis in hearts were observed in the model group, but not in the mock group (Figure 3B). In MD and HD groups, YXX keli reversed the myocardial injury induced by DOX and the area of fibrosis in the MD group was significantly less when compared with the model group.

By considering that DOX caused heart failure mainly via inducing mitochondrial dysfunction and subsequent cardiotoxicity 22, we further analyzed ATP synthesis and mitochondrial DNA mass. In left ventricular tissue, total ATP synthesis was significantly decreased in the model group compared with the mock group, and expectedly, MD and HD of YXX keli improved ATP levels (Figures 3C,D). Notably, DOX-induced decrease in mitochondrial DNA mass was also increased in MD and HD groups. Notably, Benazepril failed to affect ATP level and mitochondrial DNA mass indicating it may act via a different mechanism. There are no obvious differences between MD and LD groups in these terms; thus, the middle dose was employed for further investigation.

**FIGURE 4:**
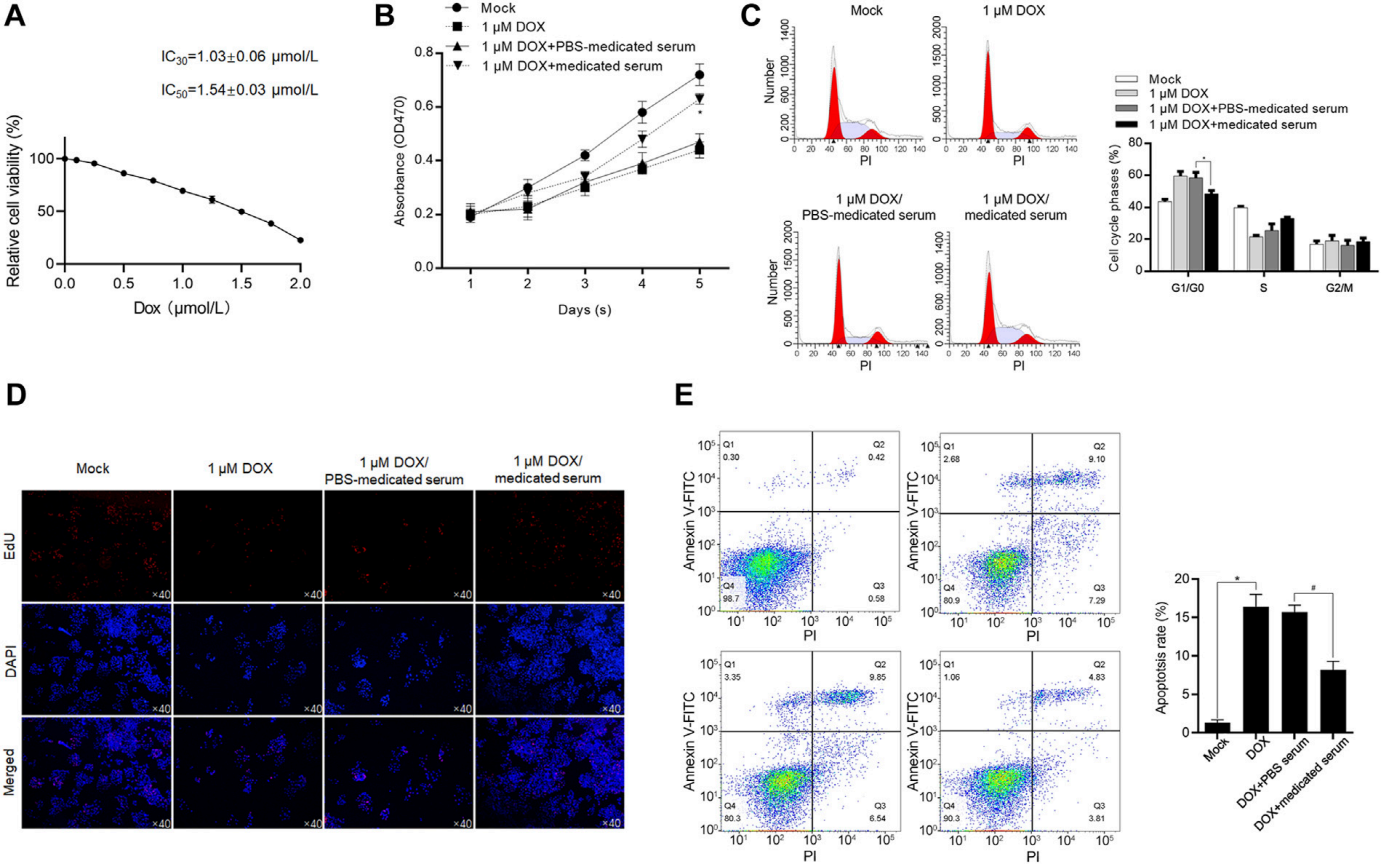
YXX keli protects RCM against DOX-induced cell injury. **(A)**. After 24-h incubation under a range concentration of DOX, the cell viability of H9c2 was measured by performing a CCK-8 assay. After the addition of medicated serum for co-incubation with 1 μM of DOX for 24 h, cell viability was measured by performing CCK-8 assay **(B)**, cell cycle distribution was measured by performing PI staining followed by flow cytometry **(C)** and proliferating cells were stained using EdU assay **(D)**. *p < 0.05, vs. 1 μM DOX + PBS-medicated serum group. **(E)** After the addition of medicated serum for co-incubation with 1.54 μM of DOX for 24 h, cell apoptosis was measured by performing Annexin V-FITC/PI double staining followed by flow cytometry assay. *p < 0.05, mock group; ^#^ *p* < 0.05, DOX + PBS-medicated serum.

YXX keli-medicated serum exerts protective effects against DOX-induced cardiac injury in H9c2 and primary rat cardiac myocytes (RCMs): with the aim to reveal the effect of YXX keli on DOX-induced cardiac injury, we analyzed the effects of supplemented 10% YXX keli-medicated serum (Medicated Serum) on cell proliferation, cell cycle distribution, and apoptosis after treatment of IC30 or IC50 of DOX for 24 h (Figure 4A). Treatment of IC30 of DOX significantly decreased cell proliferation potentially via block cell cycle at G1/G0 phase (Figures 4B–D). The addition of 10% medicated serum, but not 10% PBS serum, reversed DOX-induced cell cycle arrest. 48-h treatment of IC50 of Dox-induced apoptosis, and expectedly, 24-h treatment of medicated serum decreased DOX-induced apoptosis (Figure 4E).

**FIGURE 5:**
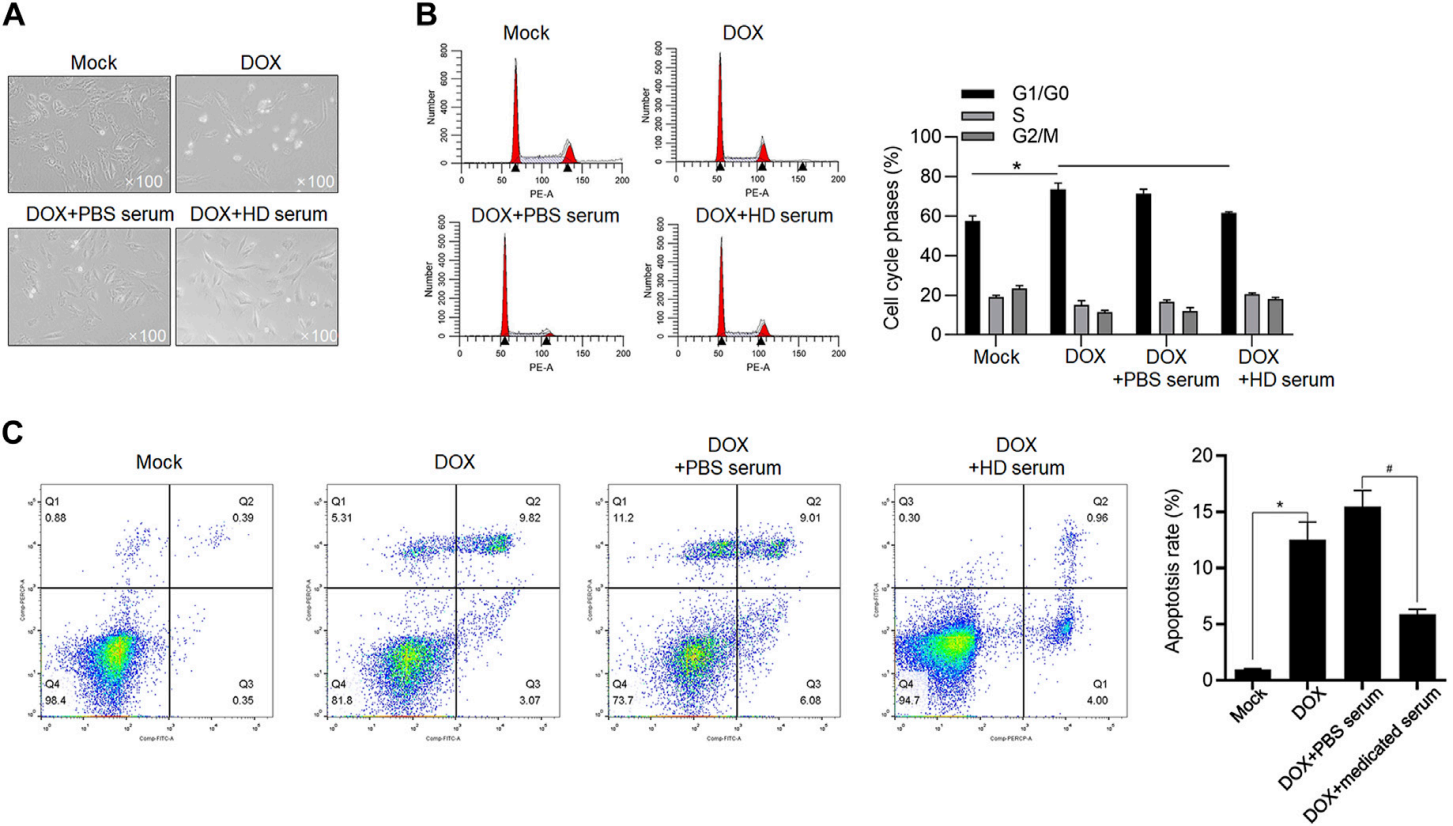
YXX keli protects cardiomyocytes against DOX-induced cell injury. **(A)**. RCMs were isolated and imaged after 1.5 μmol/L DOX treatment. **(B)**. To evaluate the effect of medicated serum on DOX-induced cell cycle arrest, H9c2 cells were exposed to 1 μmol/L DOX for 24 h, and cell cycle distribution was measured after PI staining. *p < 0.05, mock group; ^#^ *p* < 0.05, DOX + PBS-medicated serum. **(C)**. After addition of medicated serum for co-incubation with 1.54 μM of DOX for 24 h, apoptotic cell death of H9c2 was measured by performing Annexin V-FITC/PI double staining followed by flow cytometry assay. *p < 0.05, mock group; ^#^ *p* < 0.05, DOX + PBS-medicated serum.

To further confirm the protective effects of medicated serum on DOX-induced inhibition of cell proliferation and promotion of cell apoptosis, we isolated RCM and treated them with 1 μmol/L DOX for 24 h. As shown in Figure 5A, in DOX and DOX + PBS serum groups, the morphology of RCM appeared to be stick-like shape, and the addition of medicated serum obviously increased cell number and promoted cell attachment to the well plate. To further evaluate the effect of DOX on cell cycle distribution, H9c2 cells were cultured with 1 μmol/L DOX, or 1.54 μmol/L DOX for 24 h, and cell cycle or cell apoptosis was analyzed, respectively. As it is shown in Figures 5B,C, DOX treatment inhibited cell cycle progression and promoted cell apoptosis in H9c2 cells, and the addition of medicated serum significantly reversed these effects (Figures 5B,C).

**FIGURE 6:**
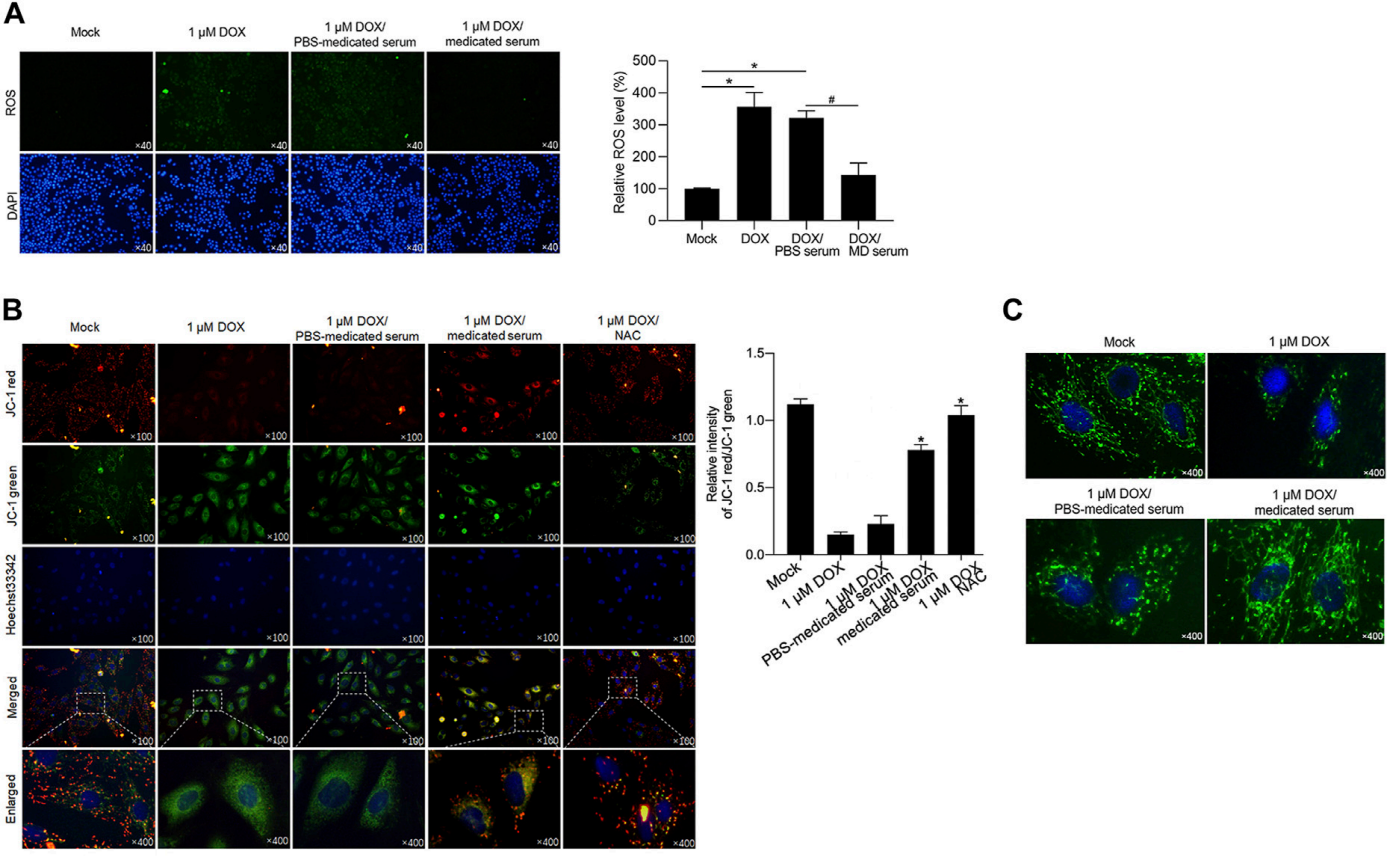
YXX keli decreased ROS accumulation induced by DOX in RCM. **(A)**. After DOX treatment, ROS accumulation was detected. *p < 0.05, vs. mock group; ^#^ *p* < 0.05, vs. DOX/PBS-medicated serum. **(B)**. Mitochondrial membrane potential was measured by performing JC-1 staining. **(C)**. Mitotracker staining was performed to detect mitochondria.

YXX keli potentially protects cardiac myocytes by decreasing DOX-induced ROS and maintaining mitochondrial homeostasis and function: DOX-induced cardiotoxicity is reported to be caused by a decrease in mitochondrial function, along with increased ROS production (Burridge et al., 2016; Maillet et al., 2016; Yoon et al., 2018). This promoted us to detect whether the addition of MD serum reverses DOX-induced mitochondrial homeostasis and dysfunction in RCM. DOX treatment for 24 h significantly increased ROS accumulation compared with the mock group (Figure 6A). An additional 24 h with the addition of MD serum, but not PBS serum, significantly decreased ROS level compared with the DOX group. By considering that ROS accumulation disrupted mitochondrial membrane potential and thus lead to mitochondrial dysfunction (Held and Houtkooper, 2015), we stained cells with JC-1, which was employed to examine mitochondrial membrane potential (Wu et al., 2018). The staining signal shows red fluorescence under normal conditions and turns green when the mitochondrial membrane potential is decreased. As shown in Figure 6B, JC-1 accumulated in functional mitochondria in the mock group. DOX-induced mitochondrial depolarization led to a change in the equilibrium of JC-1 observed as an increasing green signal and was reversed by the addition of MD serum for 24-h treatment. We further stained functional mitochondria by employing Mitotracker Green dye. As expected, DOX treatment-induced a decrease in mitochondria mass, which was reversed by the addition of MD serum (Figure 6C).

**FIGURE 7:**
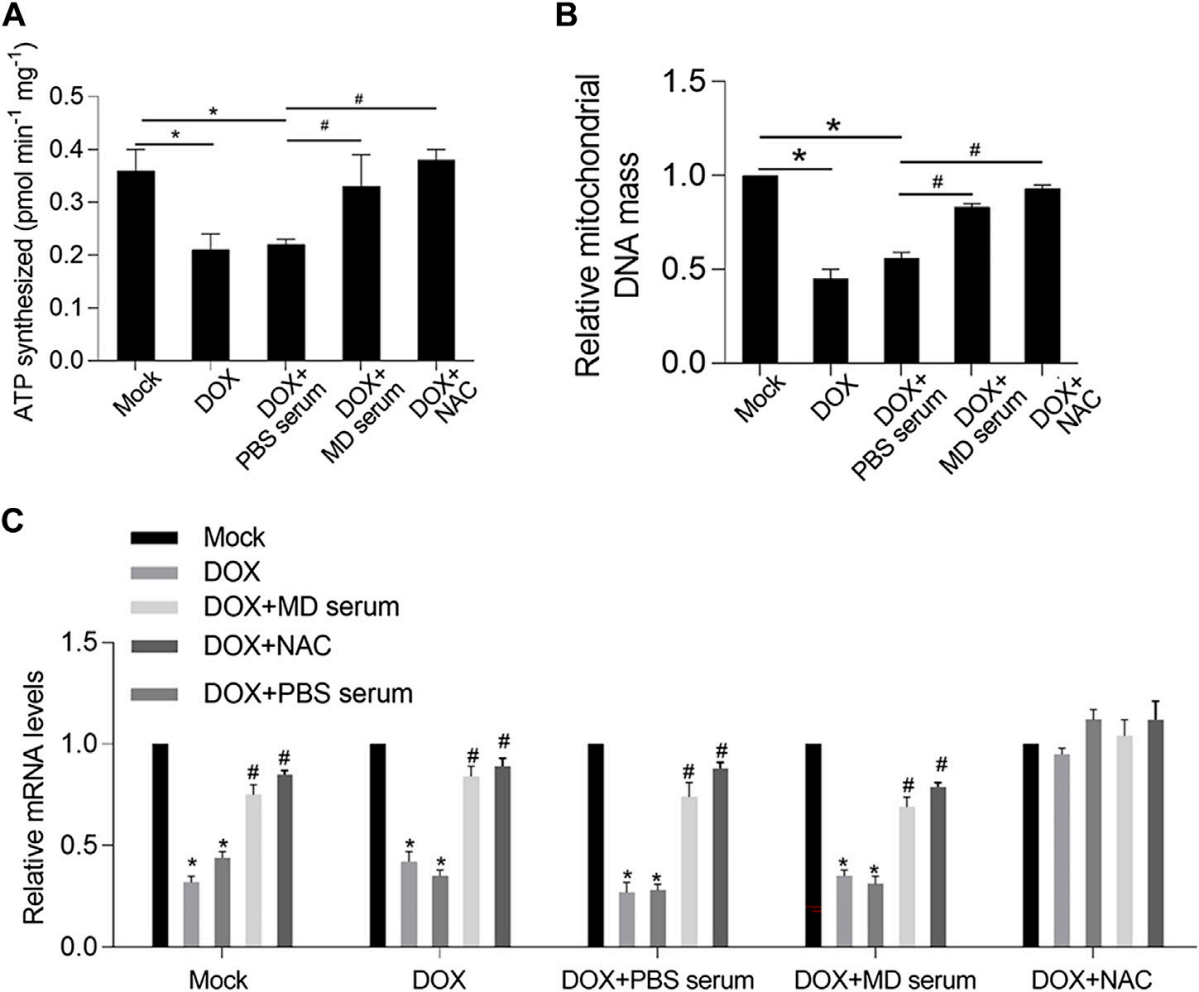
YXX keli protected mitochondrial function potentially via decreasing accumulated ROS induced by DOX in RCM. **(A)**. ATP synthesis was measured. *p < 0.05, mock group; ^#^ *p* < 0.05, DOX + PBS-medicated serum. **(B)**. Mitochondrial DNA mass was measured by quantitative PCR. *p < 0.05, mock group; ^#^ *p* < 0.05, DOX + PBS-medicated serum. **(C)**. Mitochondrial transcriptional activity was measured by performing RT-qPCR to detect the expression level of mitochondrial coded genes.

One hallmark of mitochondrial dysfunction is reduced ATP synthesis. Considering that YXX keli administration was revealed to promote ATP synthesis in the DOX-induced heart failure rat model, we wanted to determine whether the addition of MD serum could enhance ATP synthesis. Indeed, MD serum increased ATP synthesis which was decreased by DOX treatment in RCM (Figure 7A). As a positive control, NAC, a ROS scavenger, was added to erase accumulated ROS. Expectedly, scavenging of ROS induced by DOX significantly increased ATP synthesis, indicating that MD serum potentially increased ATP synthesis via scavenging ROS. We further detected mitochondrial DNA mass and transcriptional activity by detecting mitochondrial coded genes, including COX 1, COX 3, ND1, and Cyb. Both MD serum and NAC significantly increased mitochondrial DNA mass and mitochondrial transcriptional activity, which were significantly decreased by DOX (Figures 7B,C). Taken together, MD serum may increase ATP synthesis, transcriptional activity, and DNA mass of mitochondria potentially via scavenging ROS.

**FIGURE 8:**
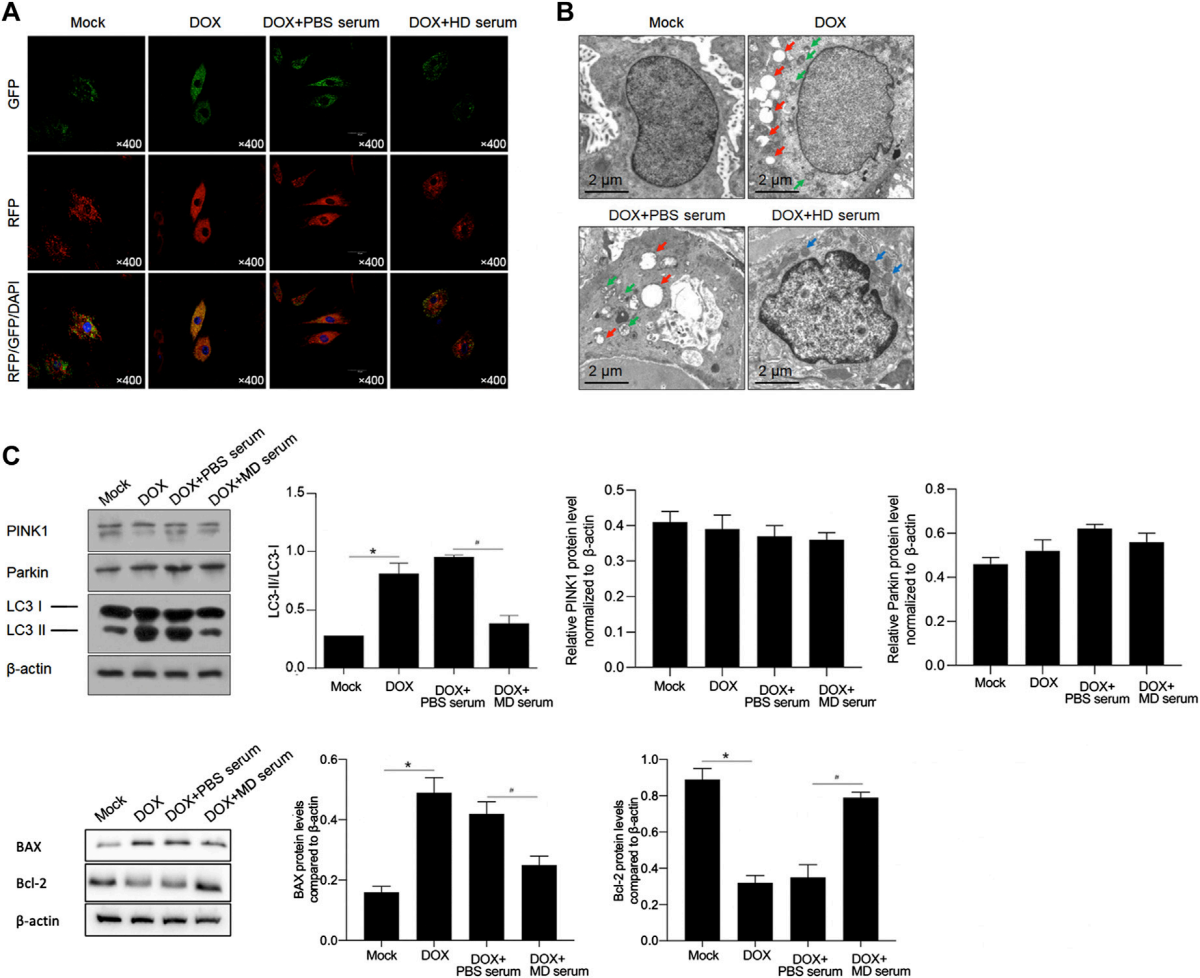
YXX keli inhibited autophagy/mitophagy in H9c2 cells. **(A)**. Formation of autophagosomes was measured by introducing GFP-RFP-LC3. **(B)**. Autophagic and mitophagic vacuoles were observed by performing electron microscopy (EM). **(C)**. Western blot was performed to detect PINK1, Parkin and cleavage of LC3 I and II. *p < 0.05, mock group; ^#^ *p* < 0.05, DOX + PBS-medicated serum. **(D)**. Western blot was further performed to detect Bax and Bcl-2 protein. *p < 0.05, mock group; ^#^ *p* < 0.05, DOX + PBS-medicated serum.

YXX keli inhibited DOX-induced autophagy/mitophagy: we next determined autophagy/mitophagy induction by the DOX treatment. By monitoring the status of autophagy/mitophagy, RFP-GFP-LC3 stable-expressing H9c2 cells were employed. Colocalization of GFP with an RFP fluorescent signal indicates an autophagosome and an RFP without GFP fluorescent signal is considered an autolysosome (Kimura et al., 2007; Li L. et al., 2014). Compared to the mock group that had minimal amounts of GFP-LC3 and RFP-LC3 puncta demonstrating a low basal level of autophagy/mitophagy, DOX treatment obviously induced the formation of autophagosomes, and the addition of MD serum obviously decreased the formation of autophagosomes (Figure 8A). Electron microscopy (EM) also confirmed that typical autophagic (red arrows) and mitophagy (green arrows) vacuoles were obviously decreased by the addition of MD serum (Figure 8B). Notably, instead of typical autophagic and mitophagy vacuoles, some abnormal mitochondria were observed, indicating that MD serum partially protects mitochondria. We further observed that MD serum significantly decreased the lapidated form of MAP1L3B/LC3B (microtubule-associated protein one light chain three beta; LC3B-II), which was increased by DOX treatment (Figure 8C). By considering that BAX and Bcl-2 are tightly involved in processes of mitophagy, we further detected BAX and Bcl-2 protein levels after medicated serum treatment. As is shown in Figure 8D, DOX treatment significantly affected BAX and Bcl-2 protein levels. The addition of MD serum further reversed the effects of DOX on both BAX and Bcl-2 protein.

## Discussion

The present study demonstrated that YXX keli improves cardiac dysfunction and metabolic processes in a rat model of DOX-induced chronic heart failure and in primary rat cardiomyocytes. In this study, heart failure and structural remodeling manifested within 8 weeks in the model group. The lumens of the hearts of mice in the model group were significantly enlarged, thus leading to decreases in EF, SV, CO (Teich), and FS, indicating cardiac dysfunction. In the model group, ATP synthesis and mitochondrial mass were significantly decreased compared to those in the control group. The administration of YXX keli significantly reversed the decreases in EF, SV, CO (Teich), and FS, which were decreased via DOX injections. Notably, ATP synthesis and mitochondrial mass were also upregulated, thus indicating the potential protective effects of YXX keli on chronic heart failure via the regulation of mitochondrial mass and its metabolic processes. In this section, and to the best of our knowledge, our valuable finding is that YXX keli exerts protective effects on DOX-induced chronic heart failure.

In numerous studies, the protective effects of TCM on CHF have been revealed, and the potential pharmacological mechanisms have also been reported (Wang Y. et al., 2017). In these studies, the DOX-induced rat cardiac toxic model was widely chosen to mimic the chronic toxic model, and the acute myocardial ischemia (AMI) rat model was chosen to mimic the acute toxic model. Therefore, the DOX-induced rat cardiac toxicity model was employed in this study. Subsequently, we also confirmed the effects of YXX keli on primary rat cardiac cells exposed to DOX. After exposure to DOX, the cellular injury was induced in primary rat cardiac cells. Due to the difficulty of culturing cardiac cells for long periods, we also employed the rat cardiac cell Line H9C2 to determine the protective effects of YXX keli on cardiac toxicity.

From *in vivo* and *in vitro* studies, TCM has been demonstrated to exert protective effects on HF *via* several properties, including antifibrosis, anti-inflammation, antioxidation, antiapoptotic, PR angiogenesis, and metabolism regulation (Wang Y. et al., 2017). Fibrosis, which causes a number of cardiovascular diseases, leads to the distortion of cardiac dysfunction. Xuefuzhuyu decoction, which is composed of 11 compounds, can decrease cardiac fibrosis induced by hypertension by decreasing TGF-β1, which is a factor contributing to myocardial fibrosis (Zhang et al., 2016). Inflammation is considered to be one of the major pathological changes in HF (Yndestad et al., 2006; Marchant et al., 2012). Qishenyiqi decoction has been reported to regulate cyclooxygenase 1 (COX 1) and COX two in the arachidonic acid (AA) pathway, which is activated by inflammation, leading to decreased fibrosis biomarkers, including matrix metalloproteinase-2 (MMP-2), MMP-9, collagen I, and collagen III (Li C. et al., 2014; Wang et al., 2015). The accumulation of ROS frequently induces oxidative stress, thus contributing to cardiac remodeling and HF. Accumulated ROS induce the loss of mitochondrial homeostasis and function, as well as the upregulation of MMPs and cardiac hypertrophy (Tsutsui et al., 2011). The Chinese herbal medicines Shanzha and Danshen have been frequently used together to treat cardiovascular diseases and exhibit high antioxidant activities (Bernatoniene et al., 2008; Tu et al., 2013; Liu et al., 2014). In this manner, accumulated ROS were scavenged by the use of Chinese herbal medicine. Meanwhile, we also observed that the addition of YXX keli exerted an inhibitory effect on autophagy/mitophagy induced by DOX by detecting LC3B, Bcl-2, and Bax. However, the addition of autophagy/mitophagy inhibitor slightly affects mitochondrial homeostasis caused by YXX keli, indicating that YXX keli may exert a regulatory role on H9C2 cells in different manners.

In this study, YXX keli was revealed to scavenge ROS after DOX exposure in H9C2 cells and primary rat cardiac cells. This result demonstrated the antioxidant properties of YXX keli upon DOX exposure. In the DOX-induced CHF rat model, we measured the ATP levels and mitochondrial DNA mass in cardiomyocytes. Expectedly, in the model group, the ATP levels and mitochondrial DNA mass were significantly decreased compared to those in the untreated group. The administration of YXX keli significantly reversed the decreases in ATP levels and mitochondrial DNA mass, indicating that the administration of YXX keli potentially reversed the decreases in mitochondrial mass and ATP synthesis. Although we failed to detect the ROS levels in these cardiomyocytes, we assumed that the regulation of ATP and mitochondrial functions by YXX keli occurred via the inhibition of oxidative stress.

## Data Availability

The original contributions presented in the study are included in the article/Supplementary Material; further inquiries can be directed to the corresponding author.
