# Supplementary figures and images for: Yang-xin-xue keli exerts therapeutic effects via regulating mitochondrial homeostasis and function in doxorubicin-induced rat heart failure
Source: Front Pharmacol. 2022 Aug 30;13:931453. doi: 10.3389/fphar.2022.931453 (PMC9468485; doi:10.3389/fphar.2022.931453)

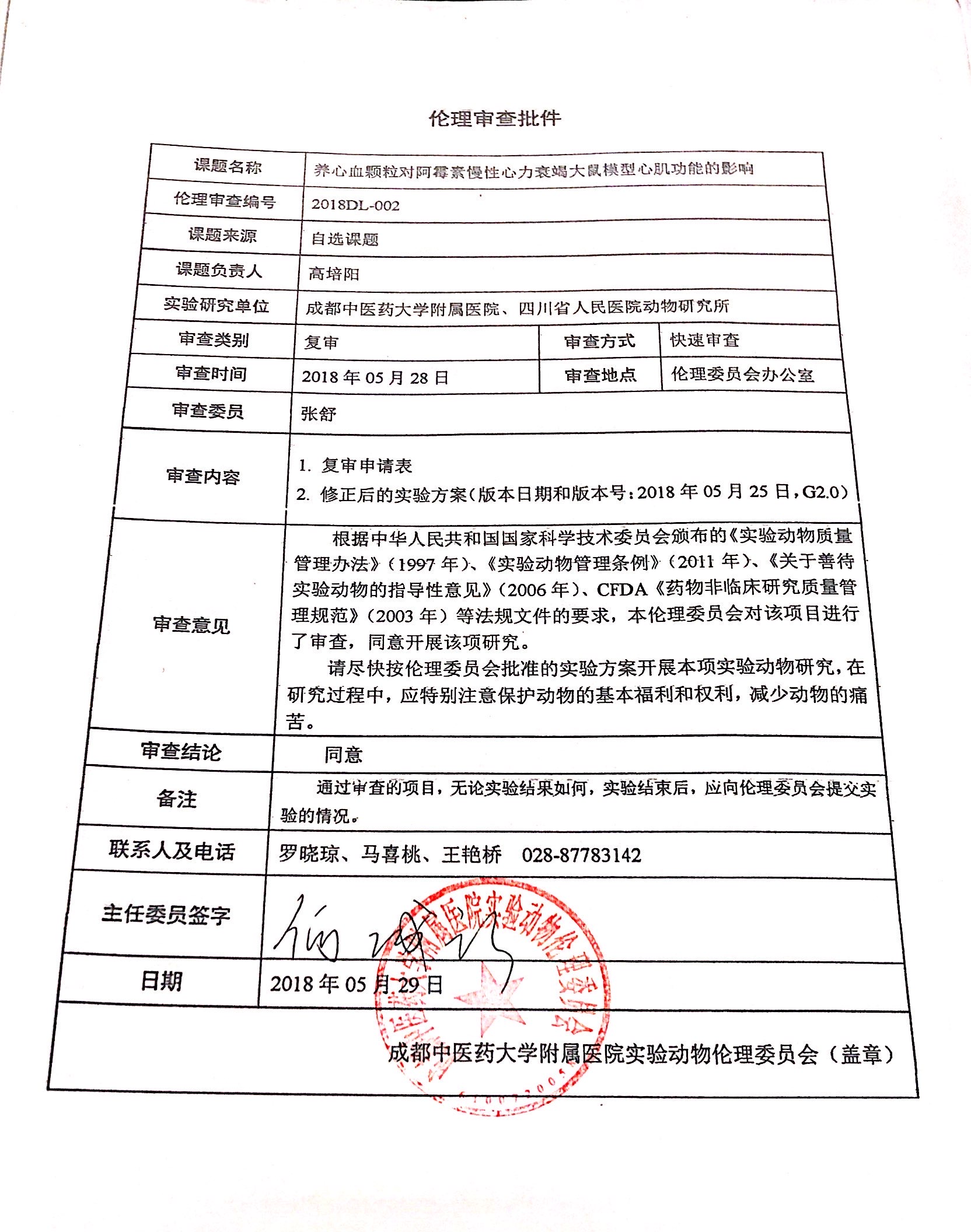

Supplement: Supplementary file 1 [file Image1.JPEG]
